# Symmetric dimethylarginine as a biomarker of renal impairment after a decade of follow-up

**DOI:** 10.1038/s41598-025-14842-y

**Published:** 2025-08-20

**Authors:** Hendrik Meyer, Marcus Dörr, Anke Hannemann, Edzard Schwedhelm, Rainer Böger, Till Ittermann, Nele Friedrich, Martin Bahls

**Affiliations:** 1 Institute of Clinical Chemistry and Laboratory Medicine, University of Greifswald, Greifswald, Germany; 2 Department of Internal Medicine B, University Medicine Greifswald, Greifswald, Germany; 3 DZHK—German Centre for Cardiovascular Research, Partner Site Greifswald, Greifswald, Germany; 4 DZHK—German Centre for Cardiovascular Research, Partner Site Hamburg/Kiel/Lübeck, Hamburg/Kiel/Lübeck, Germany; 5 Institute of Clinical Pharmacology and Toxicology, University Medical Center Hamburg-Eppendorf, Hamburg, Germany; 6 Institute for Community Medicine - SHIP Clinical-Epidemiological Research, University Medicine Greifswald, Greifswald, Germany

**Keywords:** Biomarkers, Nephrology

## Abstract

Chronic kidney disease (CKD) is characterized by a gradual and irreversible decline in renal function. Arginine derivatives like symmetric dimethylarginine (SDMA) and asymmetric dimethylarginine (ADMA) have been associated with the progression of renal disease in cross-sectional studies. This study aimed to investigate the longitudinal associations between arginine derivatives and renal function over time in a population-based cohort. Data were derived from the Study of Health in Pomerania-START (SHIP-START-0, n = 3100, mean age 49 years, women 51.3%). The 5-year follow-up included 2174 and the 10-year follow-up 1657 individuals. At baseline, serum concentrations of Arg, ADMA, SDMA and homoarginine were measured using liquid chromatography–tandem mass spectrometry, while renal parameters (creatinine and cystatin C) were quantified at baseline, 5- and 10-year follow-up. Three different statistical analyses (linear regression, logistic regression, analyses of covariance (ANCOVA)), were performed to investigate the longitudinal associations between baseline levels of Arg, ADMA, SMDA and homoarginine as exposure and renal function parameters as outcome. The results showed a significantly inverse longitudinal association between SDMA and renal function. Specifically, a one μmol/l greater SDMA was related to an odds ratio of 1.77 (95% confidence interval (CI) 1.41; 2.21) at 5-year follow-up and 1.43 (95% CI 1.11; 1.84) at 10-year follow-up for kidney dysfunction defined as estimated glomerular filtration rate less than 60 ml/min/1.73 m^2^. These findings suggest that SDMA might predict future deterioration of renal function. This supports previous findings which suggested that SDMA may be a biomarker for renal function.

**Supplementary Information:**

The online version contains supplementary material available at 10.1038/s41598-025-14842-y.

## Introduction

Chronic kidney disease (CKD) is a persistent condition marked by progressive and irreversible loss of kidney function or sustained renal damage. With an estimated 850 million individuals affected worldwide, the rising prevalence of CKD represents a significant global public health challenge^1^. CKD is a major risk factor for atherosclerosis, ultimately contributing to cardiovascular (CV) mortality, cardiomyopathy, and related complications. The initiating phases of atherosclerosis are located in the endothelial cell layer, where vascular integrity is essential for maintaining CV health. Endothelial dysfunction, which is marked by reduced nitric oxide (NO) bioavailability, is the essential initial fundamental mechanism in the pathogenesis of atherosclerosis^2^.

NO is a small gaseous signaling molecule with a key role in vascular function^3^. NO is produced from L-arginine (Arg) by the endothelial nitric oxide synthase (eNOS) in endothelial cells^4^. Symmetric (SDMA) and asymmetric dimethylarginine (ADMA) are post-translationally modified from Arg residues^5^. ADMA is an endogenous inhibitor of eNOS, thereby reducing the bioavailability of NO which leads to endothelial dysfunction. SDMA is a competitor of cellular Arg transport and is thought to indirectly inhibit eNOS by limiting the availability of Arg^6^. Serum levels of SDMA correlate well with renal function in cross-sectional settings^7,8^. Thus, SDMA is a potential marker for the estimated glomerular filtration rate (eGFR) but also for the extent of coronary artery disease^9^. Previous research already demonstrated the relationship between SDMA and renal dysfunction in several disease settings^7^. However, long-term follow-up studies in the general population are currently lacking.

Homoarginine (hArg) is a methylene homologue of L-arginine. This amino acid functions as a potential NO precursor and serves as a substrate for eNOS^10^. In contrast to ADMA and SDMA, low serum concentrations of hArg are associated with an increased risk for several diseases, especially in the CV and renal system^11,12^.

While Arg derivates are related with the progression of CV and renal diseases in cross-sectional studies with relatively small patients-based sample sizes, the association with regards to long-term risk in a population-based cohort is currently unclear. In this longitudinal study, we investigated the relation between Arg derivatives and renal function in a large population-based cohort over 5 and 10 years. We measured serum levels of Arg, hArg, ADMA and SDMA as well as surrogates of renal function. To investigate if Arg derivatives are associated with renal function, we related baseline Arg derivative concentrations with the eGFR after 5 and 10 years.

## Methods

### Study population

The presented data were derived from the population-based cohort Study of Health in Pomerania-START (SHIP-START) in West Pomerania, Germany^13^. Study design and recruitment strategy have been previously described in detail^14^. In brief, 6265 men and women (aged 20 to 79 years) were invited to the study between 1997 and 2001. At baseline 4308 subjects agreed to participate in the comprehensive examination program (response = 68.8%). The study was approved by the ethics committee of the University of Greifswald and conducted in accordance with the Declaration of Helsinki. All participants gave written informed consent. SHIP data can be applied for, for scientific and quality control purposes.

### Interview, medical and laboratory examination

A computer-aided personal interview was used to collect baseline information on socio-demographic characteristics and medical history. Smoking status was assessed by self-report and categorized into current, former, and never-smokers. Alcohol drinking habits were evaluated as beverage-specific alcohol consumption (beer, wine, and distilled spirits) and the mean daily alcohol consumption was calculated using beverage-specific pure ethanol volume proportions. Waist circumference (WC) was measured to the nearest 0.1 cm using an inelastic tape measure midway between the lower rib margin and the iliac crest in the horizontal plane, with the subject standing comfortably with weight distributed evenly on both feet. Diabetic patients were identified based on the self-reported use of antidiabetic medication [anatomic, therapeutic, and chemical (ATC) code: A10] in the last 7 days, a glycosylated hemoglobin level > 6.5% or a non-fasting glucose level > 11.1 mmol/L. Blood pressure (BP) was assessed after a 5 min resting period in sitting position. Systolic and diastolic BP were measured three times, with three minutes rest in between, on the right arm using a digital blood pressure monitor (HEM-705CP, Omron Corporation, Tokyo, Japan). The average of the second and third reading was used for analysis. Hypertension was defined by either self-reported antihypertensive medication or a systolic BP ≥ 140 mmHg and/or a diastolic BP ≥ 90 mmHg. CV disease based on a self-reported history of angina pectoris, peripheral artery disease, stroke, or myocardial infarction.

Non-fasting venous blood samples were drawn from all subjects in supine position (between 7 a.m. and 4 p.m.). Serum creatinine and cystatin C levels were measured using the Dimension Vista 1500 analytical system (Siemens Healthineers, Erlangen, Germany). Creatinine was measured enzymatically; cystatin C was measured by nephelometry.

Serum Arg, ADMA, SDMA and hArg concentrations were measured by liquid chromatography-tandem mass spectroscopy (LC–MS/MS) following established and validated protocols^11,15^. Briefly, 25 μL of serum were diluted in methanol with stable isotope labeled Arg, hArg, ADMA and SDMA. Thereafter, the guanidine compounds were converted into their butyl esters. Guanidino compound concentrations were calculated using triplicates with calibration curves based on four levels. Plate-wide quality controls (QC) were run in two levels by duplicates. A second analysis was performed on the samples to assess coefficient of variation and bias of QC, which had to be below 15%.

### Statistical analyses

Categorical data were expressed as percentages; continuous data were expressed as median (25th percentile; 75th percentile). To investigate the longitudinal associations between baseline levels of Arg, ADMA, SMDA, hArg as exposure and renal function parameters as outcomes three different analyses were performed.

In a first step, linear regression analyses with exposure parameters as continuous variable were performed. The models were adjusted for age, sex, diabetes mellitus, hypertension and WC as well as for the corresponding baseline values of the outcome variable in a second model. In a first sensitivity analysis eGFR_cyst_ and eGFR_crea/cys_ were used as exposures. In a second sensitivity analyses, we excluded all individuals without noticeable change in creatinine or cystatin C according to the reference change value (RCV). Variations in a laboratory analyte can result from analytical and biological variation. The RCV is given as: $$RCV = 2^{1/2} *Z*\left( {CV_{A}^{2} + CV_{I}^{2} } \right)^{1/2}$$ with CV_A_ = analytical impression, CV_I_ = within-subject biological variation and Z = number of standard deviations appropriate to the desired probability (in the present study Z = 1.645 for 90% significance)^16^. The biological variation for creatinine is 4.4% and for cystatin C 3.2%^17^. The mean analytical variation determined from control material in the study laboratory over the study waves for creatinine was 3.85% and for cystatin C 8.91%. The calculated RCVs were 13.6% and 22.0% for creatinine and cystatin C, respectively. In a third sensitivity analysis the main model was further adjusted for smoking, alcohol consumption, CVD (based on a self-reported history of angina pectoris, peripheral artery disease, stroke, or myocardial infarction).

In a second step, analyses of covariance (ANCOVA) also adjusted for age, sex, diabetes mellitus, hypertension and WC were calculated. For this, the exposure variables were categorized into four groups according to their quartiles. For each exposure-specific analysis a subset of individuals was selected that had matched baseline creatinine (± 5 µmol/l), cystatin C (± 0.05 mg/l) or eGFR (± 5 ml/min/1.73 m^2^) levels across all four groups. This implicates that baseline creatinine, cystatin C and eGFR levels were comparable over the categorized exposure groups.

In the third step, logistic regression analyses were performed to assess the associations between baseline levels of Arg, ADMA, SMDA or hArg and incident CKD defined as follow-up eGFR values < 50 or < 60 ml/min/1.73 m^2^. All analyses were performed for the 5-year and 10-year follow-up separately. Statistical analyses were performed using SAS version 9.4 (SAS statistical software, version 9.4, SAS Institute, Inc; NC, USA).

## Results

**Fig. 1:**
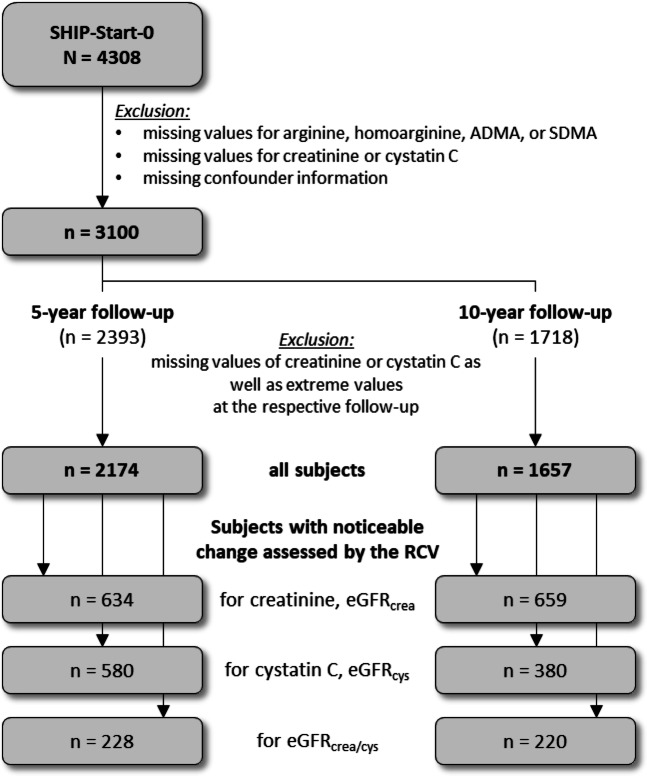
Flow diagram of the used study populations. SDMA, symmetric dimethylarginine; ADMA, asymmetric dimethylarginine, RCV, Reference Change Value; eGFR, estimated glomerular filtration rate.

Out of 4308 baseline participants, 1208 subjects were excluded due to missing values for Arg, ADMA, SDMA, hArg, creatinine and cystatin C or confounder information. For the primary analysis the eGFR was calculated based on creatine (eGFR_crea_). For sensitivity analyses eGFR was calculated using cystatin C (eGFR_cyst_) or creatine and cystatine (eGFR_crea/cys_)^18^. Out of the remaining 3100 study participants 926 were excluded at the 5-year follow-up and 517 at the 10-year follow-up due to missing or extreme values of creatinine or cystatin C. The 5-year follow-up included 2174 and the 10-year follow-up 1657 individuals. Whether changes in serial laboratory results from an individual are clinically meaningful or result from analytical or physiological variation, can be assessed using the concept of the RCV^19^. RCVs were calculated for creatinine and cystatin C as described below. In the 5-year-follow-up 634 subjects had a change larger than the RCV in creatinine and consequently in the eGFR_crea_, 580 subjects in cystatin C and the eGFR_cys_ and 228 subjects in creatinine as well as cystatin C and consequently in the eGFR_crea/cys_. In the 10-year follow-up 659 subjects had a noticeable change larger than the RCV in creatinine and the eGFR_crea_, 380 subjects in cystatin C and the eGFR_cys_ and 220 subjects in creatinine as well as cystatin C and consequently in the eGFR_crea/cys_ (Fig. 1).

The characteristics of the study population at baseline as well as creatinine, cystatin C and eGFR_crea_ at the 5-year and 10-year follow-up are shown in Table 1. Median values for creatinine and cystatin C increased slightly, while the eGFR_crea_ decreased between baseline and follow-up examinations.

**Table 1:** Characteristics of the study population.

Baseline characteristics	Study population (n = 3100)
Age, years	49 (35; 63)
Men, %	48.7
Waist circumference, cm	89 (78; 99)
Smoking, %	
Never smoker	36.0
Ex-smoker	33.9
Current smoker	30.1
Alcohol consumption, g/day	5.2 (1.3; 14.7)
HbA1c, %	5.3 (4.9; 5.7)
Diabetes mellitus, %	9.2
Systolic blood pressure, mmHg	134 (120; 148)
Diastolic blood pressure, mmHg	83 (75; 90)
Hypertension, %	50.8
History of stroke, %	2.1
History of myocardial infarction, %	3.3
Angina pectoris, %	4.2
Peripheral artery disease, %	2.7
Arginine, µmol/l	153 (121; 188)
ADMA, µmol/l	0.67 (0.59; 0.76)
SDMA, µmol/l	0.45 (0.39; 0.53)
Homoarginine, µmol/l	2.60 (2.06; 3.30)
Creatinine, µmol/l	67 (58; 77)
Cystatin C, mg/l	0.68 (0.61; 0.77)
eGFR_crea_, ml/min/1.73 m^2^	101 (89; 113)
5-year follow-up characteristics (n = 2174)	
Creatinine, µmol/l	68 (58; 78)
Cystatin C, mg/l	0.74 (0.66; 0.88)
eGFR_crea_, ml/min/1.73 m^2^	98 (85; 109)
10-year follow-up characteristics (n = 1657)	
Creatinine, µmol/l	71 (62; 82)
Cystatin C, mg/l	0.72 (0.65; 0.82)
eGFR_crea_, ml/min/1.73 m^2^	93 (80; 103)

Data are expressed as median (25th percentile; 75th percentile); nominal data are given as percentages. HbA1c, hemoglobin A1c; eGFR_crea_, estimated glomerular filtration rate based on creatinine; ADMA, asymmetric dimethylarginine; SDMA, symmetric dimethylarginine.

**Fig. 2:**
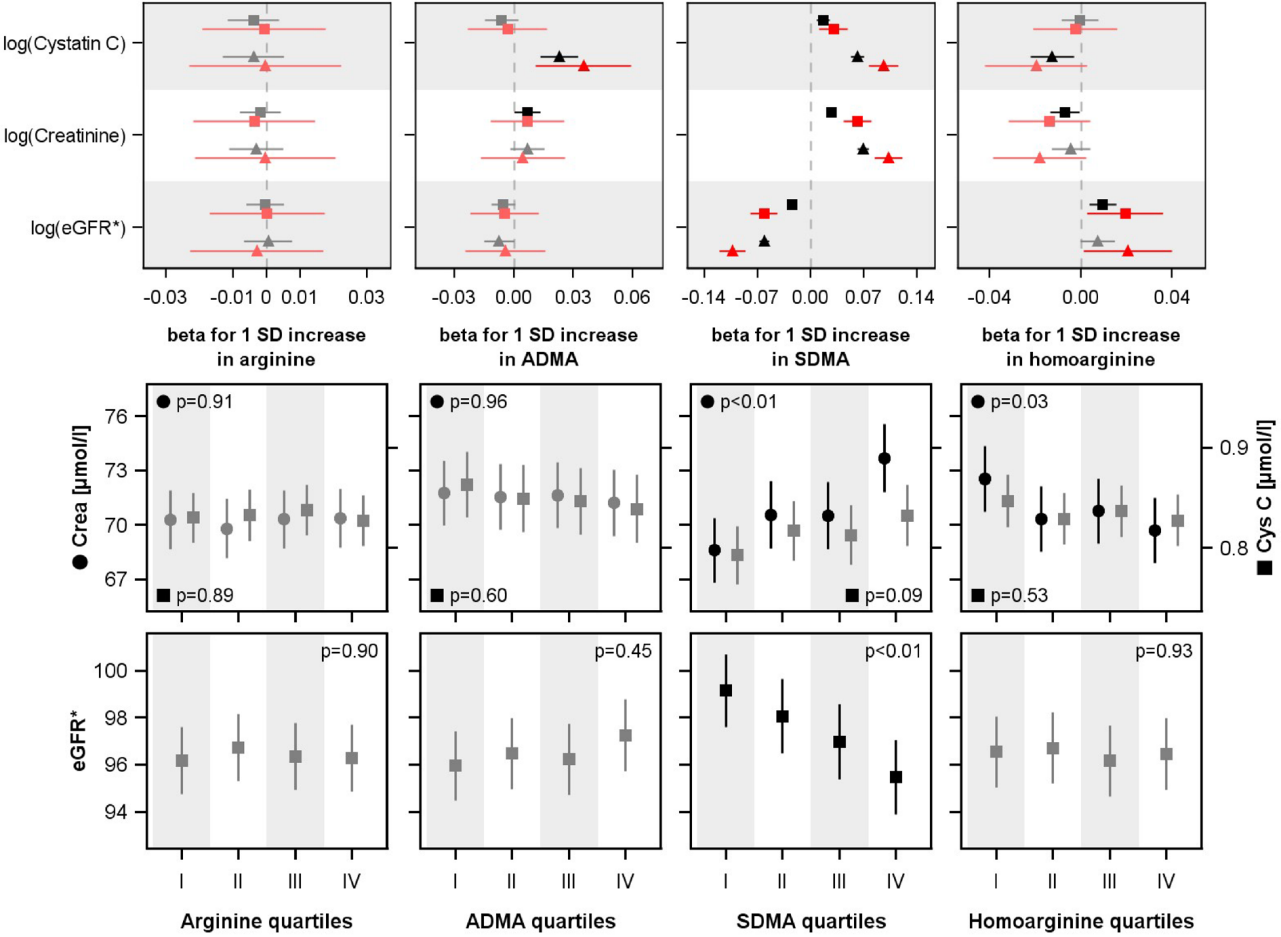
UPPER ROW. Associations of baseline arginine, ADMA and SDMA, homoarginine levels with log-transformed 5-year creatinine, cystatin C, and estimated glomerular filtration rate (eGFR_crea_) in the whole population (black) as well as in the RCV subpopulation (red). Beta coefficients with 95% confidence intervals from linear regression analyses adjusted for age, sex, diabetes mellitus, hypertension and waist circumference (triangles) and additional adjustment for baseline outcome values (squares) are illustrated. The RCV subpopulation included subjects with noticeable changes in creatinine or cystatin C between baseline and follow-up (see methods). LOWER ROW: Estimated mean levels of creatinine, cystatin C or eGFR_crea_ with 95% confidence intervals in the 5-year follow-up by quartiles of baseline arginine, ADMA and SDMA, homoarginine levels. Analyses of covariance were adjusted for age, sex, diabetes mellitus, hypertension and waist circumference. For each exposure-specific analysis a subset of individuals with matched baseline creatinine (± 5 µmol/l), cystatin C (± 0.05 mg/l) or eGFR_crea_ (± 5 ml/min/1.73 m^2^) levels across all four groups were used. SD, standard deviation; SDMA, symmetric dimethylarginine; ADMA, asymmetric dimethylarginine. *The eGFR_crea_ was calculated based on creatinine.

In the linear regression models baseline hArg and ADMA were not consistently associated with the 5-year outcome in cystatin C, creatinine or eGFR_crea_. Baseline SDMA was positively associated with creatinine and cystatin C and consequently inversely with eGFR_crea_ (Fig. 2). These results remained stable irrespective of whether the whole population or the subpopulation with noticeable changes in creatinine and cystatin C were analyzed and whether the models were adjusted for the baseline outcome value. Additional adjustment for smoking, alcohol consumption, CVD (based on a self-reported history of angina pectoris, peripheral artery disease, stroke, or myocardial infarction) did not substantially change the results (Suppl. Figure 1).

ANCOVA models confirmed the results of the linear regression models. SDMA was positively associated with the 5-year creatinine level and inversely with eGFR_crea_. We found a tendency towards a positive association between SDMA and cystatin C (*p* = 0.09). In addition, an inverse association between hArg and creatinine was found.

A logistic regression adjusted for age, sex, diabetes mellitus, hypertension and WC was used to examine the association of Arg, ADMA, SDMA or hArg with incident CKD (eGFR_crea_ < 50 ml/min/1.73 m^2^ or eGFR_crea_ < 60 ml/min/1.73 m^2^) at 5-year and 10-year follow-up. For both outcomes and both follow-up periods, the regression analyses identified significantly higher odds ratios (OR) for SDMA (all *p* ≤ 0.01; Table 2). The OR for an eGFR_crea_ < 50 ml/min/1.73 m^2^ was, for example, 2.29 (95%-CI 1.72; 3.06) with each increase in SDMA per standard deviation (SD) at the 5-year follow-up and 2.13 (95%-CI 1.51; 2.99) at the 10-year follow-up. Arg, ADMA and hArg were not consistently associated with incident CKD.

**Table 2:** Associations between arginine, homoarginine, ADMA or SDMA and incident CKD.

	eGFR < 50 ml/min/1.73 m^2^			
	5-year follow-up		10-year follow-up	
	OR (95%-CI) per SD increase	*p*	OR (95%-CI) per SD increase	*p*
N (cases)	2163 (49)		1654 (40)	
Arginine	1.21 (0.90; 1.64)	0.21	1.13 (0.79; 1.63)	0.50
ADMA	**1.34 (1.01; 1.78)**	**0.04**	1.20 (0.86; 1.66)	0.28
SDMA	**2.29 (1.72; 3.06)**	**< 0.01**	**2.13 (1.51; 2.99)**	**< 0.01**
Homoarginine	0.88 (0.61; 1.26)	0.49	1.05 (0.71; 1.53)	0.82

	eGFR < 60 ml/min/1.73 m^2^			
	5-year follow-up		10-year follow-up	
	OR (95%-CI) per SD increase	*p*	OR (95%-CI) per SD increase	*p*
N (cases)	2133 (86)		1643 (82)	
Arginine	1.12 (0.89; 1.40)	0.35	0.96 (0.74; 1.26)	0.79
ADMA	1.15 (0.92; 1.45)	0.22	0.90 (0.69; 1.17)	0.42
**SDMA**	**1.77 (1.41; 2.21)**	**< 0.01**	**1.43 (1.11; 1.84)**	**0.01**
Homoarginine	0.88 (0.67; 1.17)	0.38	0.93 (0.69; 1.25)	0.65

Logistic regression adjusted for age, sex, diabetes mellitus, hypertension and waist circumference. OR, odds ratio; CI, confidence interval; SD, standard deviation; SDMA, symmetric dimethylarginine; ADMA, asymmetric dimethylarginine; eGFR, estimated glomerular filtration rate based on creatinine.

Significant values are in bold.

**Fig. 3:**
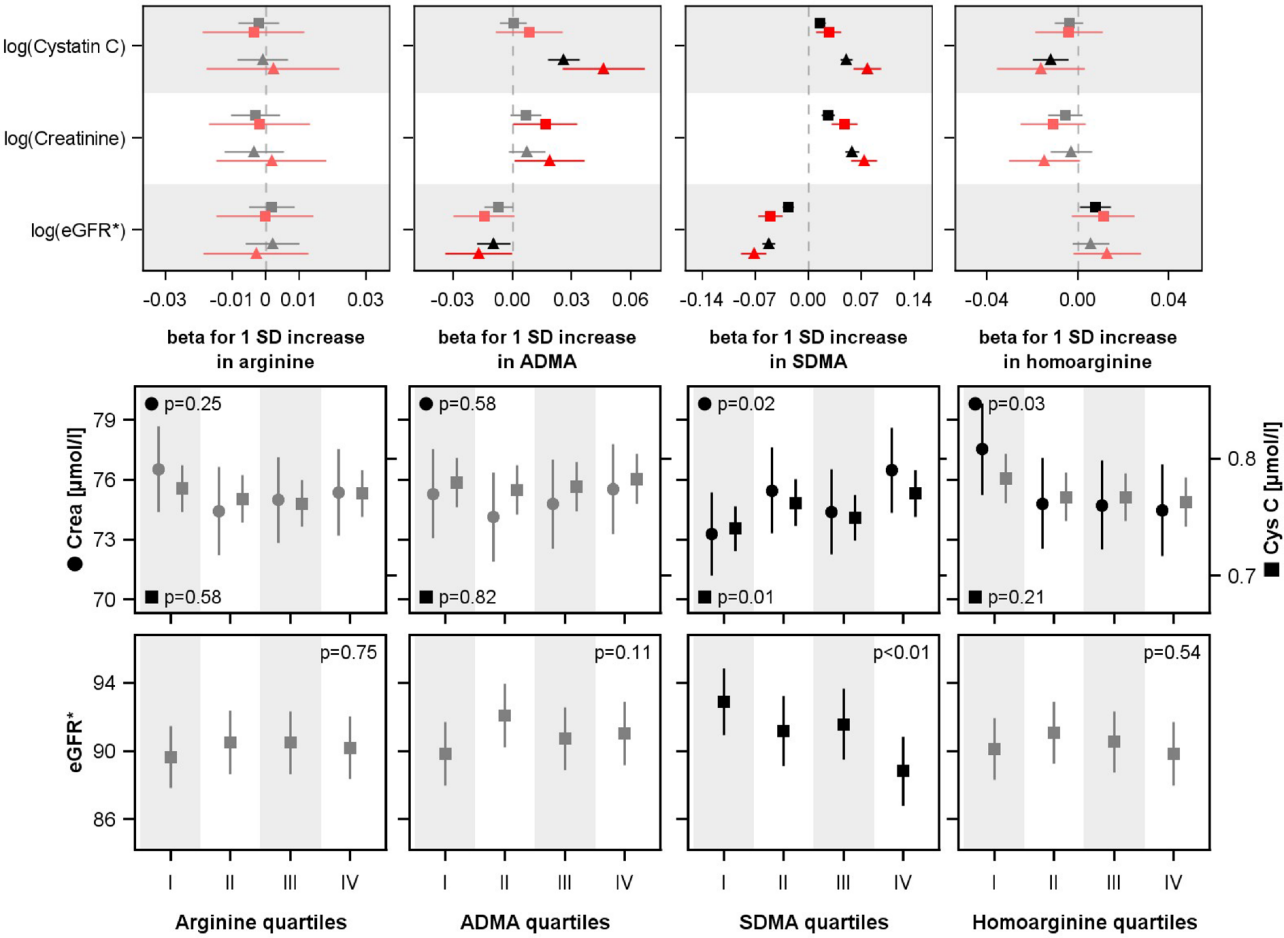
UPPER ROW. Associations of baseline arginine, ADMA and SDMA, homoarginine levels with log-transformed 10-year creatinine, cystatin C and estimated glomerular filtration rate (eGFR) in the whole population (black) as well as in the RCV subpopulation (red). Beta coefficients with 95% confidence intervals from linear regression analyses adjusted for age, sex, diabetes mellitus, hypertension and waist circumference (triangles) and additional adjustment for baseline outcome values (squares) are illustrated. The RCV subpopulation included subjects with noticeable changes in creatinine or cystatin C between baseline and follow-up (see methods). LOWER ROW: Estimated mean levels of creatinine, cystatin C or eGFR with 95% confidence intervals in the 10-year follow-up by quartiles of baseline arginine, ADMA and SDMA, homoarginine. Analyses of covariance were adjusted for age, sex, diabetes mellitus, hypertension and waist circumference. For each exposure-specific analysis a subset of individuals with matched baseline creatinine (± 5 µmol/l), cystatin C (± 0.05 mg/l) or eGFR (± 5 ml/min/1.73 m^2^) levels across all four groups were used. SD, standard deviation; SDMA, symmetric dimethylarginine; ADMA, asymmetric dimethylarginine. *The eGFR was calculated based on creatinine.

Figure 3 shows the analogue results for the 10-year follow-up. Baseline SDMA was positively associated with creatinine and cystatin C as well as inversely with eGFR_crea_. Arg showed no and hArg and ADMA no consistent associations with 10-year creatinine, cystatin C, or eGFR_crea_ after ten years. In the ANCOVA models the positive association between SDMA and creatinine or cystatin C at follow-up was confirmed. SDMA was inversely related with eGFR_crea_. Also, hArg remained inversely associated with creatinine at follow-up after ten years.

The results of the sensitivity analysis using eGFR_cyst_ and eGFR_crea/cys_ are presented in suppl. Figure 2 and suppl. Table 1. At the 5- and 10-year follow-up, higher SMDA levels were associated with CKD defined as eGFR_cyst_/eGFR_crea/cys_ < 50 or < 60 ml/min/1.73 m^2^ independent of the eGFR formula used (Suppl. Table 1). Moreover, consistent and quite comparable strong inverse associations were found between SDMA and the eGFR_crea,_ eGFR_cyst_, and eGFR_crea/cys_.

## Discussion

This study investigated the relationship between Arg, ADMA, SDMA and hArg with changes in renal function over five and ten years in a large population-based cohort. We found positive associations between baseline SDMA and creatinine as well as cystatin C levels in the 5- and 10-year follow-up. Moreover, SDMA was inversely associated with the eGFR calculated from creatinine and/or cystatin C using three different equations. Finally, higher SDMA baseline was related to greater odds for incident CKD.

Circulating levels of ADMA are controlled by their release from methylated proteins, renal function and degradation by dimethylarginine dimethylaminohydrolase (DDAH). Previous research already demonstrated that in patients with kidney disease the reduction in DDAH is more important for circulating ADMA levels compared to kidney function. Our findings also support previous findings which reported a close link between SDMA and eGFR^20^.

Our findings indicate that SDMA is associated with longitudinal changes in renal function which agrees with previous cross-sectional studies^7,8^. A biological explanation for our finding is that SDMA is excreted exclusively from the kidney. Hence, renal dysfunction results in higher concentrations of circulating SDMA^5,21^. High SDMA levels are thought to play an important role in progression of end-stage renal disease in CKD patients^22^. In fact, SDMA is a more consistent predictor of CKD progression and atherosclerotic cardiovascular events than other methylarginines in a study group including non-dialysis patients with kidney disease: Improving global outcomes (KDIGO) grades G2-G4^23^.

The structural isomer of SDMA, ADMA, is an endogenous inhibitor of NO synthase and was found to accumulate in renal failure^24^. This accumulation contributes to endothelial dysfunction eventually resulting in atherosclerosis. Higher levels of ADMA are linked to an increased CV disease risk and mortality in patients with end stage renal disease^25,26^. In our analyses, ADMA was not consistently associated with a higher creatinine or cystatin C concentration or a lower eGFR, respectively. In contrast to SDMA, ADMA is mainly eliminated by enzymatic degradation. The degrading enzyme, DDAH, is located in the kidney. Albeit, a meta-analysis reported that ADMA was not correlated with renal function, a dysfunctional kidney may also have less functional DDAH resulting in higher concentrations of circulating ADMA^7^. Nonetheless, the importance of ADMA for CV risk has been evaluated thoroughly. In a meta-analysis of 22 prospective studies, ADMA concentrations in the highest tertile were related with a 42%, 39% and 60% higher risk for CV disease, coronary artery disease and stroke, respectively^27^.

In the Dallas Heart Study hArg was inversely associated with subclinical vascular disease and risk for CVD events^28^. Further, lower levels of circulating hArg are related with impaired cardiac function and a dilated left ventricle^11^. In patients undergoing coronary angiography or hemodialysis, low hArg levels are a strong risk factor for CV and all-cause mortality^29^. In the present study, we found no consistent associations between hArg and renal function.

Although this study is based on a large and regional representative sample of the population living in northeastern Germany, our findings are limited due to a cohort of largely Caucasian decent. Hence, it remains unclear whether the results are comparable to other ethnicities. In addition, individuals with severe kidney dysfunction may not participate in SHIP or they deceased between baseline and follow-up. We also did not include potential pharmaceutical options which may influence arginine derivatives and/or renal function. Given that hArg is largely influenced by diet, we also need to advise that we have no information on the dietary habits of the study participants which may have influenced the hArg levels. However, the relatively large number of individuals in this cohort in a longitudinal study underline our findings.

To conclude, we found a consistent significant association between SDMA and changes in renal function calculated using three different formulas for eGFR. SDMA thus seems to be a suitable marker for evaluating renal function in general, hence presenting a potential alternative to commonly used biomarkers, i.e. creatinine. Given that creatinine clearance has a high inter-individual variability and also depends on muscle mass, protein intake as well as age and sex, SDMA may be a suitable alternative as a surrogate for renal function^7^. In accordance with previous studies, SDMA might not only indicate progression of renal disease but also mark early stages of CKD. Consequently, patients with high SDMA serum levels may help to guide individualized therapy and prevention. However, future studies with patients are essential to determine the future role of SDMA. Additional studies are warranted to assess how medication influences the relationship between SDMA and renal function. In summary, our findings suggest that SDMA should be used more widely in a clinical setting clinically.

## Supplementary Information

Below is the link to the electronic supplementary material.

Supplementary Material 1

## Data Availability

Data from the “Study of Health of Pomerania” are available from the University Medicine Greifswald, Germany but restrictions apply to the availability of these data, which were used under license for the current study, and so are not publicly available. Data are, however, available upon reasonable request at https://transfer.ship-med.uni-greifswald.de/FAIRequest/data-use-intro and with permission of the University Medicine Greifswald.
