# Supplementary material for: Symmetric dimethylarginine as a biomarker of renal impairment after a decade of follow-up
Source: Sci Rep. 2025 Aug 20;15:30520. doi: 10.1038/s41598-025-14842-y (PMC12368228; doi:10.1038/s41598-025-14842-y)
Supplement: Supplementary file 1 — Supplementary Material 1 [file 41598_2025_14842_MOESM1_ESM.docx]

**Supplement**


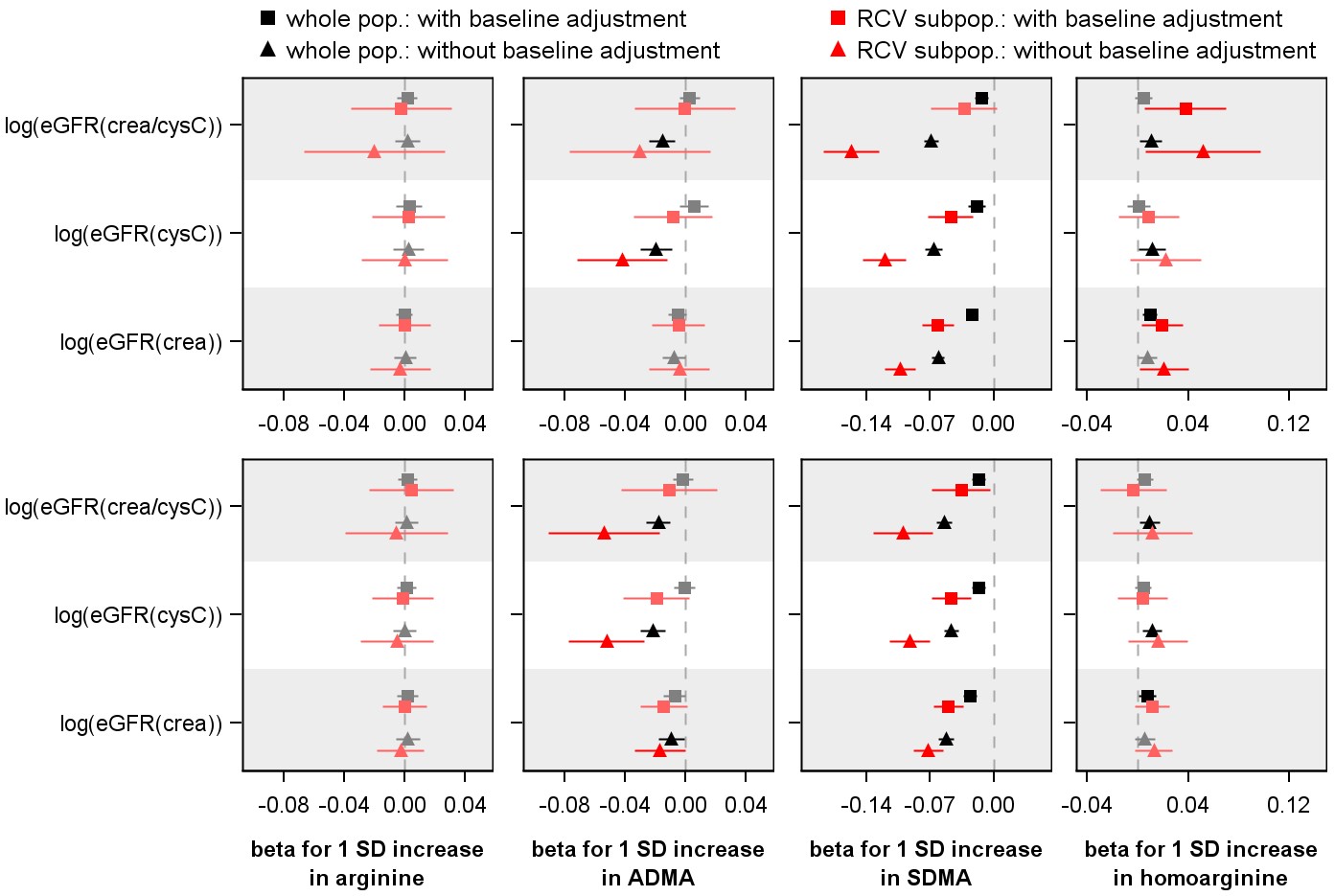


**Figure S1.** Associations of baseline arginine, ADMA and SDMA, homoarginine levels with log-transformed 5-year (upper row) or 10-year (lower row) estimated glomerular filtration rate (eGFR) values in the whole population (black) as well as in the RCV subpopulation (red). The eGFR was calculated based on creatinine, cystatin C and creatinine plus cystatin C (see methods). Beta coefficients with 95% confidence intervals from linear regression analyses adjusted for age, sex, diabetes mellitus, hypertension and waist circumference (triangles) and additional adjustment for baseline outcome values (squares) are illustrated. The RCV subpopulation included subjects with noticeable changes in creatinine or cystatin C between baseline and follow-up (see methods).

**Table S1.** Association between levels of arginine, homoarginine, ADMA or SDMA and incident CKD by using different eGFR equations.

|  | **eGFR < 50 ml/min/1.73m²** | | | | |  | **eGFR < 60 ml/min/1.73m²** | | | | |
| --- | --- | --- | --- | --- | --- | --- | --- | --- | --- | --- | --- |
|  | **5-year follow-up** | |  | **10-year follow-up** | |  | **5-year follow-up** | |  | **10-year follow-up** | |
|  | **OR (95%-CI) per SD increase** | **p** |  | **OR (95%-CI) per SD increase** | **p** |  | **OR (95%-CI) per SD increase** | **p** |  | **OR (95%-CI) per SD increase** | **p** |
|  | **eGFR_crea_** | | | | | | | | | | |
| N (cases) | 2,163 (49) |  |  | 1,654 (40) |  |  | 2,133 (86) |  |  | 1,643 (82) |  |
| Arginine | 1.21 (0.90; 1.64) | 0.21 |  | 1.13 (0.79; 1.63) | 0.50 |  | 1.12 (0.89; 1.40) | 0.35 |  | 0.96 (0.74; 1.26) | 0.79 |
| Homoarginine | 0.88 (0.61; 1.26) | 0.49 |  | 1.05 (0.71; 1.53) | 0.82 |  | 0.88 (0.67; 1.17) | 0.38 |  | 0.93 (0.69; 1.25) | 0.65 |
| ADMA | **1.34 (1.01; 1.78)** | **0.04** |  | 1.20 (0.86; 1.66) | 0.28 |  | 1.15 (0.92; 1.45) | 0.22 |  | 0.90 (0.69; 1.17) | 0.42 |
| SDMA | **2.29 (1.72; 3.06)** | **<.01** |  | **2.13 (1.51; 2.99)** | **<.01** |  | **1.77 (1.41; 2.21)** | **<.01** |  | **1.43 (1.11; 1.84)** | **0.01** |
|  |  |  |  |  |  |  |  |  |  |  |  |
|  | **eGFR_cys_** | | | | | | | | | | |
| N (cases) | 2,163 (87) |  |  | 1,652 (30) |  |  | 2,149 (151) |  |  | 1,649 (55) |  |
| Arginine | 1.04 (0.83; 1.31) | 0.71 |  | 1.30 (0.85; 2.00) | 0.22 |  | 1.04 (0.87; 1.23) | 0.69 |  | 0.98 (0.71; 1.35) | 0.90 |
| Homoarginine | 0.95 (0.74; 1.23) | 0.71 |  | 0.76 (0.46; 1.25) | 0.28 |  | 1.02 (0.84; 1.23) | 0.87 |  | 0.69 (0.46; 1.01) | 0.06 |
| ADMA | 1.03 (0.82; 1.29) | 0.83 |  | 1.16 (0.78; 1.73) | 0.47 |  | 1.09 (0.92; 1.30) | 0.32 |  | 1.24 (0.92; 1.68) | 0.16 |
| SDMA | **1.69 (1.36; 2.09)** | **<.01** |  | **2.19 (1.47; 3.27)** | **<.01** |  | **1.51 (1.28; 1.79)** | **<.01** |  | **1.95 (1.43; 2.66)** | **<.01** |
|  |  |  |  |  |  |  |  |  |  |  |  |
|  | **eGFR_crea/cys_** | | | | | | | | | | |
| N (cases) | 2,165 (65) |  |  | 1,654 (31) |  |  | 2,151 (104) |  |  | 1,651 (63) |  |
| Arginine | 1.25 (0.96; 1.62) | 0.10 |  | 1.18 (0.78; 1.78) | 0.43 |  | 1.09 (0.88; 1.35) | 0.43 |  | 1.06 (0.78; 1.44) | 0.71 |
| Homoarginine | 0.93 (0.69; 1.25) | 0.63 |  | 0.93 (0.59; 1.47) | 0.77 |  | 0.87 (0.67; 1.12) | 0.27 |  | 0.89 (0.63; 1.25) | 0.48 |
| ADMA | 1.14 (0.88; 1.48) | 0.32 |  | 1.18 (0.81; 1.73) | 0.40 |  | 1.12 (0.90; 1.38) | 0.31 |  | 1.11 (0.83; 1.49) | 0.48 |
| SDMA | **2.23 (1.73; 2.89)** | **<.01** |  | **2.45 (1.65; 3.63)** | **<.01** |  | **1.87 (1.52; 2.32)** | **<.01** |  | **1.65 (1.24; 2.21)** | **<.01** |

Logistic regression adjusted for age, sex, diabetes mellitus, hypertension and waist circumference. OR = odds ratio; CI = confidence interval; SD = standard deviation; SDMA = symmetric dimethylarginine; ADMA = asymmetric dimethylarginine; eGFR = estimated glomerular filtration rate.
